# The Effects of pH and Temperature on Cysteine Protease (Cathepsin B) Activity in Miracidia and Eggs of *Fasciola hepatica*

**Published:** 2020

**Authors:** Leila KIANIFARD, Mohammad YAKHCHALI, Mehdi IMANI

**Affiliations:** 1. Department of Pathobiology, Faculty of Veterinary Medicine, Urmia University, Urmia, Iran; 2. Department of Basic Sciences, Faculty of Veterinary Medicine, Urmia University, Urmia, Iran

**Keywords:** Cysteine proteases, Miracidia, Eggs, *Fasciola hepatica*

## Abstract

**Background:**

Fascioliasis is a worldwide zoonotic disease caused by the trematodes *Fasciola hepatica* in humans and animals. Proteases are essential for the survival of parasites and have important activities such as penetration, tissue migration, and egg hatching. This study was conducted to analyze cysteine protease of the miracidia and eggs of *F. hepatica*, and to assess the effects of pH and temperature on the proteases activity and stability.

**Methods:**

Adults *F. hepatica* were isolated from infected livers and were morphologically identified in 2018. Eggs collected from the adults and incubated in distilled water at 28 °C for 16 d to produce miracidia. The extract was collected from miracidia and eggs. A substrate for cathepsin B (Z-Arg-Arg-Pna) was used to assess the enzyme activity at different (2–12) pH levels. After homogenization, protein level was measured with Bradford method. Estimation of optimum temperature and pH was performed in the temperature range of 10–90 ° C and pH values from 2–12.

**Results:**

The highest activity of the miracidia and eggs enzyme extracts for Z-Arg-Arg-pNA was at pH 4. The miracidia extract was most stable at neutral pH and the eggs extract was most stable in acidic pH. The optimum temperature activity for both stages was 40 °C. These proteases were stable up to 40 °C.

**Conclusion:**

Upon the importance of pH and temperature in the life cycle of *F. hepatica*, the current findings can be used for induction of some modifications in pH and preventing the activity of the enzymes for decrement of the efficacy of miracidia penetration into the intermediate snails and egg hatching of this zoonotic parasite.

## Introduction

Fascioliasis is a worldwide disease caused by the digenean trematodes *Fasciola hepatica* and *F. gigantica* in human being and animals (1). WHO reported that fascioliasis is a major food-borne disease with up to 17 million human cases in 61 countries and 180 million at risk for fascioliasis (2, 3). Fascioliasis is also known as a common parasitic infection that leads to significant loss in growth and meat production in livestock in Europe, Africa, Asia, the Americas and Australasia and annual economic losses of more than US$3 billion (3).

Proteolysis is a crucial activity for many physiological operations (4). Cysteine proteases are the important enzymes that play important role in parasite invasion and survival via a range of functions that include feeding, immune evasion and modulation, tissue migration, egg production and excystment (5). They are a target for vaccine and drug production (6). *Fasciola* species express cysteine proteases such as cathepsin L and B during their life cycle (7). Cathepsin B proteases play an important role in the biology of trematode parasites. For up to 5 wk following excystment, juvenile flukes express at least three cathepsin B proteases, with FhcatB1, FhCB2, FhCB1 and FhCB3 identified as the major protein components in the ESP of *F. hepatica* (8). Cathepsin L-like proteases also reported in juvenile flukes (8). The fundamental importance of cysteine proteases in the normal functioning of parasites is highlighted by a reduction in *Schistosoma mansoni* burdens in rodent models following treatment with cysteine protease inhibitors and examples where parasite infections in vivo were eliminated using cysteine protease inhibitors (9,10). Cathepsin B might be a suitable target to reduce schistosome transmission and morbidity (7). There are several studies for the importance of cysteine proteases in liver fluke biology (11). In vitro condition, the FhCB2 is crucial in invasion through the gut wall, survival and physiological function in the NEJ form of the *F. hepatica* (12). FhCB2 is released into host tissues early after infection, is highly antigenic in animals both during infection and when injected as a vaccine and is resistant to inhibition by host cystatins (11, 13, 14). In addition, vaccination studies with cathepsin L proteases of *F. hepatica* have shown protection against challenge in rodents and ruminants (15). Cathepsin B is potential target for vaccine and antihelminthics drugs for control of fascioliasis (11).

The unique features exhibited by cysteine proteases and its apparent importance for fluke biology suggested that these enzymes and related family enzymes should targeted for development of therapeutic inhibitors or by vaccination for control of fascioliasis. Environmental factors such as pH and temperature are critical for *F. hepatica* life cycle including their penetration into the aquatic snails and egg hatching (1). In miracidia, the principal function of secreted cysteine proteases may assist in tissue invasion and penetration to the intermediate host, i.e*.* lymnaeid snails.

We aimed to evaluate the cysteine proteases of the miracidia and eggs extracts of *F. hepatica* with specific substrates and to determine the optimum pH and temperature for substrates activity. The laboratory assessment of the impact of pH and temperature on the cysteine proteases activity of the miracidia and eggs extracts of *F. hepatica* can be useful in further understanding of the degree of their effects in natural environments.

## Materials and Methods

### Helminths sampling and eggs collection

Adult *F. hepatica* was isolated from naturally infected livers of the slaughtered cattle at Urmia slaughterhouse in winter 2018. The livers cut into small pieces and the adult *Fasciola* helminths were removed from the bile ducts. *F. hepatica* saved directly in an icebox during transportation to the laboratory. The isolated *Fasciola* was morphologically identified and then were examined for the presence of the eggs by microscopic inspection, then were crushed in a mortar containing 10 ml of distilled water and sieved to collect the eggs. The eggs were washed three times using 0.086% Ringer’s solution and centrifuged at 445 *×g* for 5 min to remove debris. The eggs incubated in a dark-glass bottle containing distilled water at 24 °C for 16 d. On day 16, they exposed to 100 Watts light for 6 h to stimulate miracidia release as previously described (1).

### Cysteine proteases assay

Briefly, the miracidia and eggs were homogenized by grinding in liquid nitrogen eight times separately followed by centrifugation at 16,000 *×g* for 10 min at 4 °C. The protein concentration of the supernatant was determined with the Bradford method and Bovine serum albumin (BSA) was used as standard (16). Cysteine protease (cathepsin B) was assayed using substrate Z-Arg-Arg-pNA (Sigma Aldrich, Germany) at a final concentration of 1mM in 8% (v/v) DMSO, over a pH range of 2–12 (17). The universal buffer (85 μl) (containing acetate, phosphate and borate 50 mM) and Z-Arg-Arg-pNA substrates (5 μl) were added to miracidia and eggs extracts. Appropriate blanks were run in all cases. The hydrolysis of the substrate was monitored continuously at 405 nm using an ELISA plate reader. Estimation of optimal temperature and pH were made by using a temperature range of 10 to 90 °C and pH 2–12. The thermal stability was investigated by measuring the residual activity of the enzymes after 15 min of incubation at different temperatures before substrate addition. The stability of cathepsin B protease for miracidia and eggs across a pH range 2–12 was evaluated in different periods (0, 5, 10, 15, 20, 25 min).

## Results

### The optimum pH activity and pH stability of cysteine proteases

**Fig. 1:**
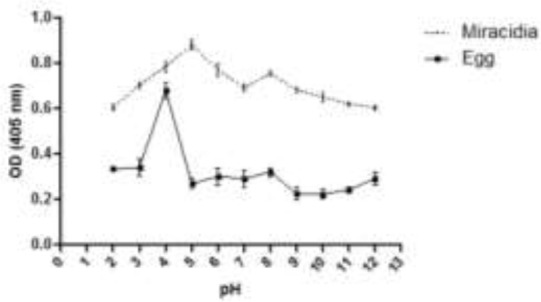
Cathepsin B activity of the miracidia and eggs extract of *Fasciola hepatica* at different pH levels by using Z-Arg-Arg-pNA substrate

Total proteins of the miracidia (2×10 ^4^ /μl) and eggs (10 ^4^ /μl) extracts were 0.471±0.0878 mg/ml and 0.233±0.0376 mg/ml, respectively. The content of the miracidia and eggs extracts revealed cathepsin B activity at pH 5 by using Z-Arg-Arg-pNA substrate. Nonetheless, pH 4 and pH 6 exhibited 89.1% and 87.4% of activity, respectively (Fig. 1). The eggs extract had the highest activity toward Z-Arg-Arg-pNA substrate at pH 4 (Fig. 1).

**Fig. 2:**
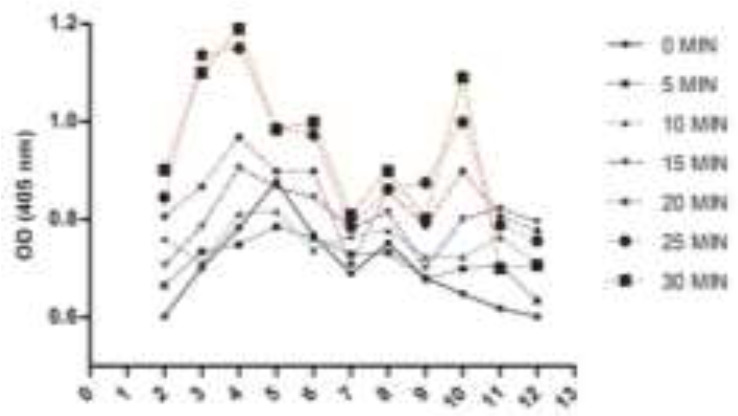
The stability of cathepsin B in the miracidia extract of *Fasciola hepatica* at pH 2–12 at different time

The miracidia extract showed greater activity compared to the eggs extract at the range of pH. The stability of cathepsin B in the miracidia extract was stable at pH 7 for 10 min incubation. It was almost stable in the pH range from 3–9. Then, stability declined as time increased from 15–30 min (Fig. 2).

**Fig. 3:**
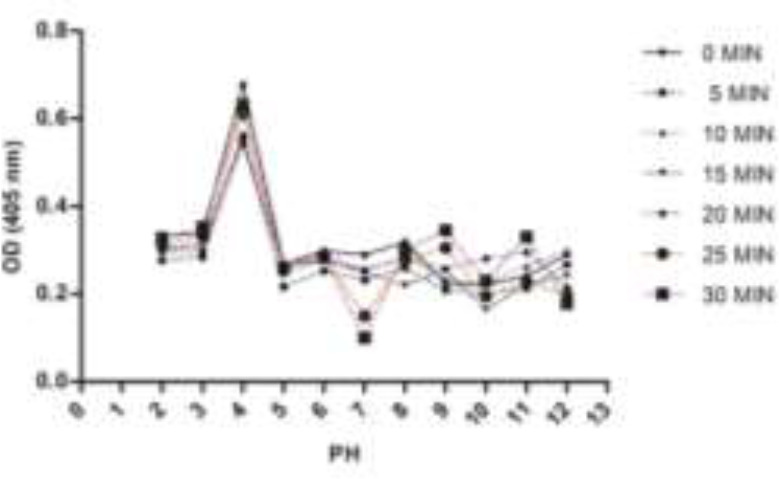
The stability of cathepsin B in the eggs extract of *Fasciola hepatica* at pH 2–12 and different times

In the eggs extract, it was most stable in an acidic environment, with optimum stability at pH 5 and 6 (Fig. 3). The enzyme stability in the eggs extract was more than miracidia.

### The optimum temperature activity and temperature stability of cysteine protease

**Fig. 4:**
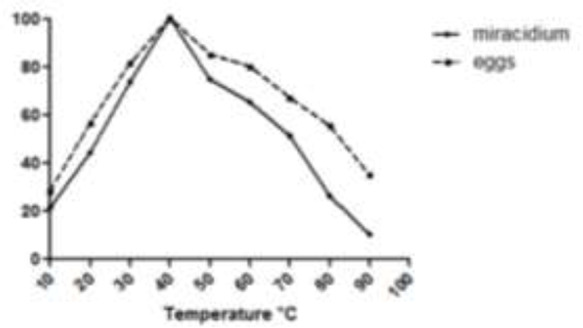
The optimum temperature of cathepsin B activities of the miracidia and eggs extracts of *Fasciola hepatica*

Fig. 4 shows the optimum temperature curves for the miracidia and eggs cathepsin B protease activities.

**Fig. 5:**
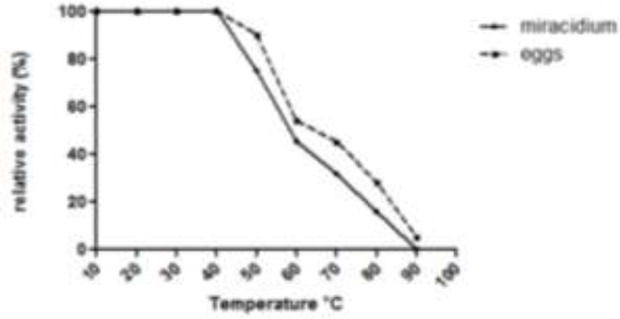
Temperature stability of cathepsin B activities of the miracidia and eggs extracts of *Fasciola hepatica*

The miracidia and eggs cathepsin B had the same optimum temperature at 40 °C, where the activity of the miracidia cathepsin B quickly declined more than the activity of the eggs cathepsin B with increasing of temperature. The effect of temperature on the stability of the miracidia and eggs cathepsin B was examined (Fig.5). The protease was stable up to 40 °C in both stages, followed by a decrease in activity with increasing of the temperature. The eggs cathepsin B had also more resistance toward the high temperatures compared to the miracidia cathepsin B.

## Discussion

Cysteine proteases play an important role in the biology of parasitic organisms. They have been found in all studied organisms with many biological phenomena and host-parasite interactions (8, 18). Cysteine proteases are a major group of proteins and their maximum activity is within a slightly acidic pH (19). The findings of this study showed that cysteine protease (cathepsin B) activities exist in the miracidia and eggs extracts of *F. hepatica* by using specific substrates Z-Arg-Arg-pNA. Cathepsin B has been previously reported in adults, juvenile and metacercariae of *F. hepatica* (8, 20, 21).

In the current study, the highest activity toward Z-Arg-Arg-pNA substrate for the miracidia and eggs extracts was at acidic pH and followed with a significant drop. In earlier studies, the role of pH on protease activity in different development stages of parasites reported (22, 23). Enzymatic assays have shown that *F. hepatica* cathepsin L activity expressed in different developmental stages (22, 24). *F gigantica* cysteine protease had pH optimum at 4.5 (25). The optimum pH for *F. gigantica* cysteine protease (PII) was at 5.5 (26). *F. hepatica* cathepsin L-like proteases were active over the pH range 5.0–9.0, with maximum activity at pH 8.0 (18). Our findings showed that the miracidia cathepsin B is stable at neutral pH and the eggs cathepsin B at acidic pH. Dowd reported that *F. hepatica* cathepsin L was stable in the pH range 5.5–9.0 and remained fully active up to 20 h at 37 °C, pH 7.0, and retained 90% of its activity after 72 h (18).

Rat liver cathepsin L was only 7% active following one-hour incubation at 37 °C (27). Turk et al. measured the temperature stability of mammalian cathepsin L and found that the rate of inactivation increased when the incubation temperature of the enzyme increased at 5–37 °C (28). The stability of cathepsin B in the miracidia extract was most stable at pH 7 for the 10 min. In the eggs extract, it was most stable in the acidic environment, with optimum stability at pH 5 and pH 6. The results of the current study indicated that the miracidia and eggs cathepsin B had the same optimum temperature activity at 40 °C. When the temperature increased, the activity cathepsin B quickly declined in miracidia. The protease was stable up to 40 °C in both stages, followed by a decrease in activity with increasing of the temperature. The eggs cathepsin B had more resistance toward the high temperatures compared to the miracidia cathepsin B. The lower optimal temperature (28 °C) reported for serine protease from *Leishmania amazonensis* and the thermal stability indicated that 50% of the enzymatic activity preserved after 4 min of pre-treatment at 42 °C and after 24 h of temperature at 37 °C (29). The stability study of *F. hepatica* cathepsin L _1_ demonstrated the enzyme retained 100% of its activity at 37 °C for 24 h (18). *Eimeria tenella* serine proteases were stable at room temperature for 24 h and storage at −20 °C for 3 months (30). The dependence of enzymatic activity on environment temperature reported for proteases of *Acanthamoeba healyi*, which causes granulomatous encephalitis and pneumonitis (31).

According to the importance of pH and temperature in the life cycle of *F. hepatica*, determination of the optimum pH and temperature activity for cathepsin B in the miracidia and eggs of this parasite may help us for prevention and controlling of fascioliasis. In miracidia, the principal function of cathepsin B might be assistance in parasite invasion and migration into snails’ body, while eggs cysteine proteases play a role in excystment and egg hatching, rather than other roles (32, 33).

## Conclusion

Knowing the optimal pH, temperature, and stability of the enzyme can be used for alteration of pH and temperature probably in reducing miracidia penetration into the intermediate snails, and egg hatching.
